# The economic costs of excessive sedentary behaviour in Japan

**DOI:** 10.1093/pubmed/fdag029

**Published:** 2026-04-24

**Authors:** Seigo Mitsutake, Ai Shibata, Kaori Ishii, Neville Owen, Koichiro Oka

**Affiliations:** Human Care Research Team, Tokyo Metropolitan Institute for Geriatrics and Gerontology, 35-2 Sakae-cho, Itabashi-ku, Tokyo 173-0015, Japan; Waseda Institute for Sport Sciences, Waseda University, 2-579-15 Mikajima, Saitama 359-1192, Japan; Institute of Health and Sport Sciences, University of Tsukuba, 1-1-1 Tennodai, Ibaraki 305-8577, Japan; Faculty of Sport Sciences, Waseda University, 2-579-15 Mikajima, Saitama 359-1192, Japan; School of Health Sciences, Swinburne University of Technology, Hawthorn, Melbourne 3122, Victoria, Australia; Physical Activity Laboratory, Baker Heart & Diabetes Institute, 75 Commercial Road, Melbourne 3004, Victoria, Australia; Faculty of Sport Sciences, Waseda University, 2-579-15 Mikajima, Saitama 359-1192, Japan

**Keywords:** chronic disease, health impact assessment, physical activity

## Abstract

**Background:**

Estimating the economic costs of excessive sedentary behaviour is essential for bridging the gap between policy development and implementation. We estimated economic costs of chronic diseases associated with excessive sedentary behaviour among Japanese adults.

**Methods:**

The prevalence-based and population-attributable fraction approaches were used to estimate the economic costs of excessive sedentary behaviour (≥8 hours of daily sitting) with Japanese national datasets in 2021. Direct healthcare and indirect costs and total costs for seven chronic diseases, such as cardiovascular diseases, associated with excessive sedentary behaviour were estimated.

**Results:**

The total costs of chronic diseases due to excessive sedentary behaviour were ~¥282.5 billion (95% CI: ¥258.9–¥306.0 billion). The total direct healthcare costs of chronic diseases due to excessive sedentary behaviour were ~¥238.4 billion (95% CI: ¥215.3 billion–¥261.5 billion) and the indirect costs were ¥44.1 billion (95% CI: ¥24.3 billion–¥61.0 billion). Outpatient direct healthcare costs were highest for diabetes, whereas inpatient direct healthcare costs were highest for dementia.

**Conclusions:**

This study highlights the importance of addressing excessive sedentary behaviour, which could contribute to reducing the healthcare costs for highly prevalent chronic diseases and ultimately to a more sustainable healthcare system in Japan.

## Introduction

With the rapid growth of ageing populations, the prevention of chronic diseases has become a public health concern around the world.^1^^,^^2^ Chronic diseases impose a heavy burden on patients and healthcare systems.^1^ The global cost of chronic diseases is projected to reach $47 trillion by 2030.^3^ Improving individual lifestyle factors that lead to chronic diseases is crucial for achieving a sustainable healthcare system in ageing societies.^1^^,^^4^

Excessive sedentary behaviour, defined as spending 8 hours or more sitting a day, is one of the major risk factors for multiple chronic diseases among adults.^5–9^ Appropriate interventions could reduce sedentary behaviour and help prevent chronic diseases.^10^ According to the World Health Organization guideline on physical activity and sedentary behaviour, meeting the recommendations of this guideline could result in significant healthcare cost savings.^11^ However, there is limited evidence on healthcare cost savings through reducing excessive sedentary behaviour. A recent systematic review found that only a few studies had examined healthcare costs associated with excessive sedentary behaviour at the national level.^12^

Economic analysis is essential for bridging the gap between policy development and implementation, increasing political engagement, and motivating actions.^13^ Several studies using national data have estimated the economic costs of excessive sedentary behaviour.^13–17^ For example, in Western countries, excessive sedentary behaviour has been shown to impose substantial social and economic costs related to chronic diseases.^14–17^ In the UK, direct healthcare costs for chronic diseases due to excessive sedentary behaviour were estimated at £0.8 billion in a single financial year.^16^ In these studies, aggregated healthcare costs, including outpatient and inpatient healthcare costs, have been reported.^14–17^ However, few studies have separately estimated the outpatient and inpatient healthcare costs attributable to excessive sedentary behaviour. Estimating these costs separately could better inform policymakers’ decisions on healthcare resource allocation (e.g. hospital bed capacity or primary care provider capacity), because healthcare resource use and care pathways differ between outpatient and inpatient healthcare.^18^ Furthermore, as this disaggregation aligns with international healthcare cost accounting standards,^19^ estimates from Japan can provide internationally comparable evidence.

No studies have estimated the economic costs of the excess amounts of sedentary behaviour at the national level in East Asian countries. Because Japan is one of the most aged countries, with approximately 30% of its population aged 65 years or older, information on the economic costs of excessive sedentary behaviour in Japan may help policymakers in other ageing societies.^20^ Intervention to reduce excessive sedentary behaviour is crucial for a sustainable healthcare system in ageing societies, because sedentary time and the associated risk of chronic diseases and their complications tend to increase with age.^21–23^ In addition, the recent public physical activity guidelines in Japan have primarily focused on moderate-to-vigorous physical activity, with less focus on excessive sedentary behaviour.^24^ According to Japanese government statistics, in FY 2021, much of the medical care expenditure (excluding prescription medications) was accounted for by chronic diseases led by circulatory diseases (¥6116 billion), followed by neoplasms (¥4828 billion), within a total of ¥32 403 billion.^25^ Estimating the economic costs of chronic diseases associated with excessive sedentary behaviour may help to develop/revise the guidelines for sedentary behaviour in Japan. The current study estimated the direct healthcare and indirect costs of chronic diseases associated with excessive sedentary behaviour in Japan.

## Methods

### Study design

This study used a prevalence-based cost-of-illness design with a population-attributable fraction (PAF) approach to estimate the annual direct and indirect costs of chronic diseases associated with excessive sedentary behaviour in Japan.

### Analytical approach

The economic costs attributed to excessive sedentary behaviour were estimated using three key information sources: relative risk (RR) estimates of the chronic diseases associated with excessive amounts of sedentary behaviour, the prevalence of excessive sedentary behaviour among Japanese adults, and the direct healthcare and indirect costs of the chronic diseases associated with excessive sedentary behaviour.^13–17^

Excessive sedentary behaviour was defined as ≥8 hours per day of sitting, based on prior studies.^5–9^ According to the Japanese National Health and Nutrition Survey,^26^ 35.3% of Japanese adults are classified as having excessive sedentary behaviour (men: 38.1%; women: 32.8%) (Supplemental Table 1). Direct healthcare, indirect, and total costs were estimated for seven chronic diseases associated with excessive sedentary behaviour—cardiovascular diseases (CVDs), diabetes, colon cancer, lung cancer, endometrial cancer, dementia, and depression—selected based on prior studies.^14–17^ Direct healthcare costs and indirect costs for these diseases could be estimated from publicly available Japanese national datasets.

Direct healthcare and indirect costs for each chronic disease due to excessive sedentary behaviour were calculated based on the approach of prior studies using PAF.^13–17^^,^^27^ The PAF (%) estimates the contribution of excess amounts of sedentary behaviour to the direct healthcare cost and indirect cost of each chronic disease due to the excessive time spent in sedentary behaviour. All the costs were calculated using the JPY (1€ = ¥129.88 in 2021).^28^ From the meta-analyses, the RRs for chronic diseases associated with excessive sedentary behaviour were identified (Table 1).^5–9^^,^^29^

**Table 1:** PAF for chronic diseases associated with excessive sedentary behaviour (≥8 hour per day of sedentary time) by gender in Japanese adults.

Chronic diseases	Reference	RR (95% CI)	PAF, % (95% CI)		
			Total	Men	Women
CVD	Jingjie *et al.*	1.24 (1.21–1.27)	6.8 (6.1–7.5)	7.4 (6.6–8.1)	6.3 (5.7–7.0)
Diabetes	Bailey *et al.*	1.11 (1.01–1.19)	3.5 (0.3–5.6)	3.8 (0.4–6.1)	3.3 (0.3–5.2)
Colon cancer	Ma *et al.*	1.06 (1.03–1.09)	2.0 (1.0–2.9)	2.2 (1.1–3.1)	1.9 (1.0–2.7)
Lung cancer	Shen *et al.*	1.27 (1.06–1.52)	7.5 (2–12.1)	8.1 (2.2–13.0)	7.0 (1.9–11.2)
Endometrial cancer	Shen *et al.*	1.28 (1.08–1.53)	7.7 (2.6–12.2)	8.3 (2.8–13.2)	7.2 (2.4–11.4)
Dementia	Yan *et al.*	1.30 (1.12–1.51)	8.1 (3.8–11.9)	8.8 (4.1–12.9)	7.6 (3.5–11.1)
Depression	Zhou *et al.*	1.42 (1.22–1.67)	10.4 (6.4–14.2)	11.3 (6.9–15.3)	9.7 (5.9–13.2)

Abbreviations: PAF, population attributable fraction; CVD, cardiovascular disease; RR, relative risk; CI, confidence interval.

The PAF (%) combines the RR estimate with the population prevalence (*P*_1_) of excessive amounts of sedentary behaviour using the below formula^15^^,^^17^^,^^30^:


$$\mathrm{PAF}\ \left(\%\right)=\frac{P_1\ \left(\mathrm{RRs}-1\right)}{\mathrm{RRs}}\times{100}_{.}$$

The total direct healthcare cost (outpatient and inpatient direct healthcare), indirect cost, and total economic costs were multiplied by each PAF (%) and the 95% confidence interval (CI) separately by sex to estimate the economic cost due to the excessive time spent in sedentary behaviour.

### Direct healthcare costs

Direct healthcare costs, including outpatient direct healthcare, inpatient direct healthcare, and total direct healthcare costs, among people aged 15 or older (Supplemental Table 2) in the period 1 April 2021 to 31 March 2022 [financial year (FY) 2021] were identified using the Japanese Estimates of National Medical Care Expenditure.^25^ The Japanese Estimates of National Medical Care Expenditure have healthcare costs of outpatient care including home medical care and inpatient care related to chronic diseases separated by sex and age group, but do not include healthcare costs related to medications prescribed. The healthcare cost of each chronic disease was classified using the International Classification of Diseases, 10th Revision (ICD-10) codes in the Japanese Estimates of National Medical Care Expenditure: CVD (I20–I25); diabetes, including type I and II (E10–E14); colon cancer (C18–C20); lung cancer (C33–C34); endometrial cancer (C53–C55); dementia, including Alzheimer’s disease (F01, F03, G30); and depression, including the other mood disorders (F30–F39).

### Indirect costs

Indirect costs were estimated using lost productivity due to premature mortality because of chronic diseases associated with excessive sedentary behaviour in the period 1 January 2021 to 31 December 2021.^14^^,^^31^ Indirect costs were calculated by sex using the number of deaths due to eligible chronic diseases by sex and age group from the Japanese Vital Statistics (Supplemental Table 3),^32^ the present value of potential lifetime earnings by sex and age group based on future earnings from the Japanese Basic Survey on Wage Structure lost to the present values with a discount rate of 3% according to the WHO guide to cost,^20^^,^^33^ and the average employment rate by sex and age group from the Japanese Labour Force Survey.^31^^,^^34^ The number of deaths due to chronic diseases was identified using the same ICD-10 codes as those classifying healthcare costs in the Estimates of National Medical Care Expenditure.

## Results

In Japanese adults, the PAF (%) values for excessive sedentary behaviour in the total population were lowest for colon cancer (2.0%; 95% CI: 1.0%–2.9%) and highest for depression (10.4%; 95% CI: 6.4%–14.2%) among chronic diseases (Table 1). The PAF (%) values were between 2.2% (colon cancer) and 11.3% (depression) among men and between 1.9% (colon cancer) and 9.7% (depression) among women.

The total direct healthcare costs of chronic diseases associated with excessive sedentary behaviour were ¥238.4 billion (€1.84 billion) (95% CI: ¥215.3–¥261.5 billion) [inpatient direct healthcare cost: ¥131.1 billion (€1.01 billion) (95% CI: ¥119.0–¥143.1 billion) and outpatient direct healthcare cost: ¥107.3 billion (€0.83 billion) (95% CI: ¥94.6–¥120.0 billion)] (Table 2). Among the total population, the total direct healthcare costs of CVD due to excessive sedentary behaviour were the highest [¥46.6 billion (€0.36 billion) (95% CI: ¥41.8–¥51.2 billion)], outpatient direct healthcare costs for diabetes associated with excessive sedentary behaviour were the highest [¥32.6 billion (€0.25 billion) (95% CI: ¥3.3–¥52.5 billion)], and inpatient direct healthcare costs for dementia were the highest [¥34.0 billion (€0.26 billion) (95% CI: ¥15.8–¥49.7 billion)].

**Table 2:** Direct healthcare costs (total, inpatient, and outpatient healthcare cost) of chronic diseases associated with excessive sedentary behaviour (≥8 hour per day of sedentary time) in Japanese adults.

Disease	Direct healthcare cost, ¥ billion^a^ (95% CI)		
	Total	Men	Women
Total direct healthcare cost			
CVD	46.6 (41.8–51.2)	35.6 (31.9–39.1)	12.6 (11.3–13.9)
Diabetes	41.8 (4.2–67.4)	26.4 (2.6–42.5)	16.1 (1.6–26.0)
Colon cancer	11.9 (6.1–17.4)	7.5 (3.9–10.9)	4.6 (2.4–6.7)
Lung cancer	45.5 (12.1–73.2)	32.9 (8.8–52.9)	13.9 (3.7–22.4)
Endometrial cancer	8.1 (2.8–12.9)	0.0 (0.0–0.0)	7.5 (2.6–12.0)
Dementia	45.9 (21.3–67.1)	17.3 (8–25.2)	27.8 (12.9–40.6)
Depression	38.6 (23.5–52.4)	16.3 (9.9–22.1)	21.8 (13.3–29.6)
Total	238.4 (215.3–261.5)	136.0 (122.0–150.0)	104.4 (94.2–114.7)
Inpatient healthcare cost			
CVD	32.0 (28.7–35.1)	25.2 (22.6–27.7)	8.0 (7.1–8.8)
Diabetes	9.2 (0.9–14.9)	5.2 (0.5–8.4)	4.1 (0.4–6.6)
Colon cancer	7.5 (3.9–11.0)	4.7 (2.4–6.8)	3.0 (1.5–4.4)
Lung cancer	24.8 (6.6–39.9)	17.8 (4.7–28.6)	7.7 (2.1–12.4)
Endometrial cancer	5.4 (1.8–8.5)	0.0 (0.0–0.0)	5.0 (1.7–7.9)
Dementia	34.0 (15.8–49.7)	13.8 (6.4–20.2)	19.7 (9.1–28.8)
Depression	18.3 (11.1–24.8)	7.0 (4.3–9.5)	10.9 (6.7–14.8)
Total	131.1 (119.0–143.1)	73.7 (66.9–80.5)	58.4 (52.5–64.2)
Outpatient healthcare cost			
CVD	14.6 (13.1–16.1)	10.4 (9.3–11.4)	4.7 (4.2–5.1)
Diabetes	32.6 (3.3–52.5)	21.2 (2.1–34.1)	12.0 (1.2–19.4)
Colon cancer	4.4 (2.3–6.4)	2.8 (1.5–4.2)	1.6 (0.8–2.4)
Lung cancer	20.7 (5.5–33.3)	15.1 (4.0–24.3)	6.2 (1.7–10.0)
Endometrial cancer	2.8 (0.9–4.4)	0.0 (0.0–0.0)	2.6 (0.9–4.1)
Dementia	11.9 (5.5–17.4)	3.5 (1.6–5.1)	8.1 (3.7–11.8)
Depression	20.3 (12.4–27.6)	9.3 (5.7–12.6)	10.9 (6.6–14.8)
Total	107.3 (94.6–120.0)	62.3 (54.1–70.5)	46.1 (41.1–51.1)

Abbreviations: CVD, cardiovascular disease; CI, confidence interval.

^a^1€ = ¥129.88 in 2021.

The indirect costs of chronic diseases associated with excessive sedentary behaviour were ¥44.1 billion (€0.34 billion) (95% CI: ¥24.3–¥61.0 billion) among the total population (Table 3). The indirect costs of CVD due to excessive sedentary behaviour were the highest among the total population [¥17.5 billion (€0.13 billion) (95% CI: ¥15.7–¥19.3 billion)] and among men [¥19.9 billion (€0.15 billion) (95% CI: ¥17.9–¥21.9 billion)]. Among women, the indirect costs of endometrial cancer due to excessive sedentary behaviour were the highest [¥4.9 billion (€0.04 billion) (95% CI: ¥1.6–¥7.7 billion)].

**Table 3:** Indirect costs (lost production due to premature mortality) of chronic diseases associated with excessive sedentary behaviour (≥8 hour per day of sedentary time) in Japanese adults.

Disease	Indirect cost, ¥ billion^a^ (95% CI)		
	Total	Men	Women
CVD	17.5 (15.7–19.3)	19.9 (17.9–21.9)	1.6 (1.4–1.8)
Diabetes	1.8 (0.2–2.9)	1.9 (0.2–3.0)	0.3 (0.0–0.4)
Colon cancer	4.2 (2.2–6.1)	3.3 (1.7–4.8)	1.1 (0.6–1.6)
Lung cancer	12.2 (3.2–19.6)	11.4 (3.0–18.3)	2.3 (0.6–3.7)
Endometrial cancer	7.7 (2.6–12.2)	0.0 (0.0–0.0)	4.9 (1.6–7.7)
Dementia	0.2 (0.1–0.4)	0.2 (0.1–0.3)	0.1 (0.0–0.1)
Depression	0.3 (0.2–0.5)	0.3 (0.2–0.4)	0.1 (0.1–0.1)
Total	44.1 (24.3–61.0)	37.0 (23.1–48.8)	10.2 (4.4–15.3)

Abbreviations: CVD, cardiovascular disease; CI, confidence interval.

^a^1€ = ¥129.88 in 2021.

The total economic costs of chronic diseases due to excessive sedentary behaviour among the total population, men and women, were ¥282.5 billion (€2.18 billion) (95% CI: ¥258.9–¥306.0 billion), ¥173.0 billion (€1.33 billion) (95% CI: ¥158.5–¥187.5 billion), and ¥114.7 billion (€0.88 billion) (95% CI: ¥104.3–¥125.0 billion), respectively (Table 4). The total economic costs of CVD due to excessive sedentary behaviour were highest among the total population [¥64.1 billion (€0.49 billion) (95% CI: ¥61.7–¥66.6 billion)]. In addition, the total economic cost of CVD was the highest for men [¥55.6 billion (€0.43 billion) (95% CI: ¥53.6–¥57.6 billion)] and the total economic cost of dementia was the highest for women [¥27.8 billion (€0.21 billion) (95% CI: ¥21.4–¥34.2 billion)].

**Table 4:** Total costs (direct healthcare costs and indirect costs) of chronic diseases associated with excessive sedentary behaviour (≥8 hour per day of sedentary time) in Japanese adults.

Disease	Total cost, ¥ billion^a^ (95% CI)		
	Total	Men	Women
CVD	64.1 (61.7–66.6)	55.6 (53.6–57.6)	14.2 (13.6–14.9)
Diabetes	43.6 (30.8–56.4)	28.3 (20.2–36.3)	16.4 (11.5–21.3)
Colon cancer	16.1 (13.2–19.0)	10.8 (8.9–12.7)	5.7 (4.6–6.8)
Lung cancer	57.7 (43.3–72.0)	44.3 (33.7–54.9)	16.2 (11.9–20.5)
Endometrial cancer	15.8 (12.6–19.1)	0.0 (0.0–0.0)	12.4 (9.8–15)
Dementia	46.1 (35.5–56.8)	17.5 (13.5–21.5)	27.8 (21.4–34.2)
Depression	39.0 (32.1–45.8)	16.6 (13.7–19.5)	21.9 (18.0–25.8)
Total	282.5 (258.9–306.0)	173.0 (158.5–187.5)	114.7 (104.3–125.0)

Abbreviations: CVD, cardiovascular disease; CI, confidence interval.

^a^1€ = ¥129.88 in 2021.

## Discussion

We identified the economic cost of chronic diseases due to excessive sedentary behaviour among Japanese adults using several national datasets. The annual total direct healthcare costs and indirect costs associated with excessive sedentary behaviour were ~¥282.5 billion (€2.18 billion). These findings emphasize the importance of developing strategies to reduce the longer periods of time spent in sedentary behaviour, which could contribute to more sustainable healthcare systems in ageing societies like Japan. In addition, the outpatient healthcare costs for diabetes associated with excessive sedentary behaviour were the highest, and the inpatient healthcare costs for dementia were the most significant. The contribution of excessive sedentary behaviour to dementia-associated healthcare costs is likely to grow in an ageing society.

We estimated that direct healthcare costs for chronic diseases associated with excessive sedentary behaviour were ~¥238.4 billion (€1.84 billion), ~0.8% of the overall direct healthcare costs among adults (¥30 734 billion) for FY 2021 in Japan.^25^ Compared to a prior Canadian study that selected similar chronic diseases for estimating healthcare costs (contributing to 1.3%–1.6% of the overall burden of illness cost for 2021),^14^ the contribution of excessive sedentary behaviour to direct healthcare costs appears smaller in our study. Although the population of Canadian adults was about one-third that of Japan (Canadian adults: ~30 million, Japanese adults: 100 million),^35^ the Canadian study reported direct healthcare costs associated with excessive sedentary behaviour of CAD 2.1 billion (€1.42 billion, based on the 2021 exchange rate, €1 = CAD 1.4826), ~80% of these healthcare costs (€1.84 billion) estimated in the current study.^14^^,^^28^ This difference may be partly due to the difference in prevalence estimates for excessive sedentary behaviour in the two studies: 75.3% for men and 80.0% for women in the Canadian study and 38.1% for men and 32.8% for women in Japan.^14^ However, a prior study using the International Physical Activity Questionnaire reported that the prevalence of excessive sedentary behaviour among Japanese adults was higher than among Canadian adults.^36^ Thus, the prevalence of excessive sedentary behaviour among Japanese adults may be underestimated in the current study. Although we used prevalence from a national survey, a recent Japanese study using a questionnaire to assess sedentary behaviour time from a national survey showed that the prevalence of excessive sedentary behaviour (25.3%) was lower than we found it to be. ^26^^,^^37^ Further studies are required to identify the prevalence of excessive amounts of sedentary behaviour among Japanese adults using valid assessment tools, enabling more accurate estimation of healthcare costs from excessive sedentary behaviour from national datasets.

The current study showed that across diseases, CVD generated the most significant costs attributable to excessive sedentary behaviour in the total population, for both direct healthcare costs and indirect costs. Despite differences in disease selection, cost components (e.g. direct costs only), and exposure sedentary thresholds (e.g., ≥6 hours per day) across prior national studies from Canada, the UK, France, and Finland, this rank ordering is consistent with their findings.^14–17^ Given that sedentary behaviour and physical inactivity are established risk factors for CVD,^38^ reducing excessive sedentary time is likely to lower both direct and indirect costs. While several prior studies combined inpatient and outpatient care data when estimating national healthcare costs,^14–17^ the current study is the first to separately evaluate the contribution of excessive sedentary behaviour to these costs. In Japan, for FY 2021, the breakdown of direct healthcare costs was ~52% for inpatient care and 48% for outpatient care. However, we found that the outpatient care percentage is slightly lower, with inpatient care accounting for 57% and outpatient care for 43%. This discrepancy may be attributed to not including renal diseases (e.g. end-stage renal disease or dialysis)—which typically result in increasing outpatient care costs related to chronic conditions—as a chronic disease.^25^^,^^39^ In addition, outpatient healthcare costs for diabetes due to excessive sedentary behaviour were the highest, whereas the primary contributor to inpatient healthcare costs was dementia. Although the present finding that diabetes is the chronic disease associated with excess amounts of sedentary behaviour that has the highest cost burden is consistent with the findings of previous studies,^14–17^ the current study is the first to identify dementia as generating the highest inpatient healthcare costs. In the Canadian study, dementia contributed substantially to hospital care costs, but CVD and cancer contributed more than dementia.^14^ Dementia is known to elevate hospitalization costs due to longer hospital stays and complications during hospitalization.^40^^,^^41^ Additionally, ageing significantly increases the prevalence of dementia.^42^ The difference between the Canadian study and our study may be due to the higher proportion of older adults aged 65 or older in Japan than in Canada.^14^ In 2021, the percentage of older adults was higher in Japan than in Canada, at 28.9% of the population in Japan compared with 19.0% in Canada.^43^^,^^44^ The contribution of excessive sedentary behaviour related to dementia to healthcare costs will grow in ageing societies.

Although the findings of this study emphasize the importance of developing initiatives to prevent excessive sedentary behaviour in ageing societies, few interventions to reduce the time spent in sedentary behaviour have shown cost-effectiveness.^45^ Future studies are needed to examine the effects on healthcare costs of interventions to reduce excessive sedentary behaviour.^12^^,^^45^ Specifically, analysing the effects of such interventions on outpatient and inpatient care costs separately could provide valuable insights into their impact on healthcare costs. The Japanese government has distinguished the roles of outpatient and inpatient healthcare costs: outpatient healthcare costs are regarded as outcomes of health promotion and preventive interventions for worsening diabetes, while inpatient healthcare costs are linked to promoting differentiation and collaboration of hospital functions based on the regional medical plan.^46^ The contributions of excessive sedentary behaviour to outpatient and inpatient costs would differ depending on the chronic disease, as well as the different components of outpatient and inpatient healthcare.^25^ In an ageing society, it is crucial to examine the cost-effectiveness of interventions to reduce sedentary behaviour on inpatient healthcare costs for dementia as these costs associated with excessive amounts of sedentary behaviour are likely to increase.

The strength of this study lies in its use of several national datasets in Japan and estimates of inpatient and outpatient healthcare costs separately. However, there are several limitations. First, the estimates of direct healthcare costs did not include costs related to prescription of medications and long-term care services for chronic diseases because there was no information on the costs of prescribed medications in the national dataset. In FY 2021, the percentage of costs regarding prescribed medications was ~24% of the total healthcare costs under the mandatory medical insurance system in Japan.^25^ Additionally, healthcare costs associated with health check-ups and healthcare services not covered under the mandatory medical insurance system in Japan were not included as direct healthcare costs. Therefore, the direct healthcare costs reported in this study are likely underestimated. Second, the Estimates of National Medical Care Expenditure used in this study calculated the healthcare costs associated with each chronic disease based on the primary diagnosis. Therefore, direct healthcare costs did not take into account the costs due to comorbidity, although comorbidities are common in older adults.^47^ Third, even though the current study estimated the lost productivity due to premature mortality associated with excessive sedentary behaviour as an indirect cost, productivity losses from sickness-related absences, disability pensions, the costs of institutional long-term care, and informal care from the family were not calculated. As a result, the indirect costs estimated in this study may be underestimated. Despite these limitations, the findings of the current study may provide valuable insights for policymakers in decision-making regarding healthcare resource allocation and strategies to reduce sedentary behaviour in ageing societies.

## Conclusion

The current study found that in Japan the direct healthcare costs of chronic diseases due to excessive sedentary behaviour were ~¥238.4 billion and indirect costs ¥44.1 billion in FY 2021, totalling ¥282.5 billion (€2.18 billion). In addition, although the outpatient direct healthcare costs for diabetes due to excessive sedentary behaviour were the highest, the inpatient direct healthcare costs for dementia were the most significant. These findings imply that developing a strategy to reduce excessive amounts of sedentary behaviour could contribute to developing sustainable healthcare systems in Japan. In an ageing society, healthcare costs from excessive sedentary behaviour related to dementia are likely to grow.

## Supplementary Material

fdag029_Supplemental_Files

## Data Availability

The data underlying this article can be accessed via the URLs in Supplemental Table 2.
