# Supplementary material for: The economic costs of excessive sedentary behaviour in Japan
Source: J Public Health (Oxf). 2026 Apr 24;48(2):601–9. doi: 10.1093/pubmed/fdag029 (PMC13223573; doi:10.1093/pubmed/fdag029)
Supplement: fdag029_Supplemental_Files [file fdag029_supplemental_files.zip › Supplemental_table1.docx]

| Supplemental table 1. The percentage of excessive sedentary behaviour (≥8 hours per day of sedentary time) by age group and sex among Japanese adults | | | | | | | | | |
| --- | --- | --- | --- | --- | --- | --- | --- | --- | --- |
| Age group,  years | Total | | | Men | | | Women | | |
|  | Total, n | Excessive sedentary behaviour | | Total, n | Excessive sedentary behaviour | | Total, n | Excessive sedentary behaviour | |
|  |  | n | % |  | n | % |  | n | % |
| 20-29 | 618 | 219 | 35.4 | 301 | 99 | 32.9 | 317 | 120 | 37.9 |
| 30-39 | 870 | 285 | 32.8 | 414 | 149 | 36.0 | 456 | 136 | 29.8 |
| 40-49 | 1,098 | 393 | 35.8 | 511 | 201 | 39.3 | 587 | 192 | 32.7 |
| 50-59 | 1,052 | 364 | 34.6 | 496 | 195 | 39.3 | 556 | 169 | 30.4 |
| 60-69 | 1,522 | 503 | 33.0 | 695 | 266 | 38.3 | 827 | 237 | 28.7 |
| ≥70 | 1,922 | 735 | 38.2 | 873 | 345 | 39.5 | 1,049 | 390 | 37.2 |
| Total | 7,082 | 2,499 | 35.3 | 3,290 | 1,255 | 38.1 | 3,792 | 1,244 | 32.8 |

Data source: Ministry of Health, Labour and Welfare. The National Health and Nutrition Survey in Japan, 2013. <https://www.mhlw.go.jp/bunya/kenkou/eiyou/dl/h25-houkoku.pdf> [Accessed February 18 2025]
