# Supplementary material for: The economic costs of excessive sedentary behaviour in Japan
Source: J Public Health (Oxf). 2026 Apr 24;48(2):601–9. doi: 10.1093/pubmed/fdag029 (PMC13223573; doi:10.1093/pubmed/fdag029)
Supplement: fdag029_Supplemental_Files [file fdag029_supplemental_files.zip › Supplemental_table2.docx]

| Supplemental table 2. Data sources estimating direct healthcare costs and indirect costs | | | | |
| --- | --- | --- | --- | --- |
| Variables | Data sets (data collection year) | Title | Conducted by | URL |
| Direct healthcare cost | Japanese Estimates of National Medical Care Expenditure (April 1, 2021–March 31, 2022) | Medical Care Costs and ratios: inpatient-outpatient, by age group, disease classification, and sex | Ministry of Health, Labour and Welfare | <https://www.e-stat.go.jp/stat-search/database?page=1&statdisp_id=0003355135> |
| Indirect cost | Japanese Vital Statistics (January 1, 2021–December 31, 2021) | Number of deaths of cause of death: by age group and sex | Ministry of Health, Labour and Welfare | <https://www.e-stat.go.jp/stat-search/files?page=1&layout=datalist&toukei=00450011&tstat=000001028897&cycle=7&year=20210&month=0&tclass1=000001053058&tclass2=000001053061&tclass3=000001053065&result_back=1&tclass4val=0> |
|  | Japanese Basic Survey on Wage Structure (January 1, 2021–December 31, 2021) | Cash salary amounts by age group | Ministry of Health, Labour and Welfare | <https://www.e-stat.go.jp/stat-search/files?page=1&toukei=00450091&tstat=000001011429> |
|  | Japanese Labour Force Survey (January 1, 2021–December 31, 2021) | Labour force population ratio, employment rate, and unemployment rate by age group (since 1953) | Statistics Bureau of Japan | <https://www.e-stat.go.jp/dbview?sid=0002060049> |
