# Supplementary material for: The economic costs of excessive sedentary behaviour in Japan
Source: J Public Health (Oxf). 2026 Apr 24;48(2):601–9. doi: 10.1093/pubmed/fdag029 (PMC13223573; doi:10.1093/pubmed/fdag029)
Supplement: fdag029_Supplemental_Files [file fdag029_supplemental_files.zip › Supplemental_table3.docx]

| Supplemental table 3. The number of deaths due to eligible chronic diseases from January 1, 2021 to December 31, 2021 by sex and age group | | | | | | | | | | | | | | | | | | | |
| --- | --- | --- | --- | --- | --- | --- | --- | --- | --- | --- | --- | --- | --- | --- | --- | --- | --- | --- | --- |
|  | Age group, years | | | | | | | | | | | | | | | | | | |
| Disease | 15-19 | 20-24 | 25-29 | 30-34 | 35-39 | 40-44 | 45-49 | 50-54 | 55-59 | 60-64 | 65-69 | 70-74 | 75-79 | 80-84 | 85-89 | 90-94 | 95-99 | ≥100 |  |
| CVD | |  |  |  |  |  |  |  |  |  |  |  |  |  |  |  |  |  |  |
| Total | 4 | 12 | 20 | 66 | 129 | 337 | 847 | 1,540 | 2,001 | 2,829 | 4,306 | 8,076 | 8,854 | 11,611 | 13,088 | 9,744 | 3,858 | 647 |  |
| Men | 3 | 6 | 16 | 53 | 117 | 275 | 727 | 1,327 | 1,736 | 2,384 | 3,414 | 6,109 | 5,869 | 6,834 | 6,692 | 3,959 | 1,087 | 112 |  |
| Women | 1 | 6 | 4 | 13 | 12 | 62 | 120 | 213 | 265 | 445 | 892 | 1,967 | 2,985 | 4,777 | 6,396 | 5,785 | 2,771 | 535 |  |
| Diabetes |  |  |  |  |  |  |  |  |  |  |  |  |  |  |  |  |  |  |  |
| Total | 2 | 9 | 18 | 16 | 41 | 67 | 171 | 258 | 387 | 526 | 918 | 1,727 | 1,811 | 2,505 | 2,878 | 2,138 | 741 | 140 |  |
| Men | 1 | 6 | 10 | 11 | 32 | 49 | 137 | 196 | 316 | 435 | 685 | 1,243 | 1,132 | 1,456 | 1,338 | 712 | 175 | 18 |  |
| Women | 1 | 3 | 8 | 5 | 9 | 18 | 34 | 62 | 71 | 91 | 233 | 484 | 679 | 1,049 | 1,540 | 1,426 | 566 | 122 |  |
| Colon cancer | |  |  |  |  |  |  |  |  |  |  |  |  |  |  |  |  |  |  |
| Total | 2 | 8 | 22 | 64 | 139 | 285 | 636 | 1,136 | 1,749 | 2,589 | 4,614 | 8,010 | 7,545 | 8,561 | 8,529 | 6,053 | 2,184 | 288 |  |
| Men | 1 | 4 | 13 | 34 | 78 | 143 | 379 | 649 | 1,099 | 1,696 | 3,001 | 5,236 | 4,588 | 4,684 | 3,846 | 2,047 | 534 | 44 |  |
| Women | 1 | 4 | 9 | 30 | 61 | 142 | 257 | 487 | 650 | 893 | 1,613 | 2,774 | 2,957 | 3,877 | 4,683 | 4,006 | 1,650 | 244 |  |
| Lung cancer | |  |  |  |  |  |  |  |  |  |  |  |  |  |  |  |  |  |  |
| Total | 0 | 5 | 3 | 19 | 60 | 176 | 421 | 873 | 1,608 | 3,031 | 6,577 | 13,746 | 13,978 | 14,204 | 12,431 | 6,936 | 1,889 | 251 |  |
| Men | 0 | 4 | 2 | 12 | 34 | 114 | 285 | 594 | 1,171 | 2,292 | 5,052 | 10,524 | 10,407 | 10,060 | 8,014 | 3,872 | 771 | 69 |  |
| Women | 0 | 1 | 1 | 7 | 26 | 62 | 136 | 279 | 437 | 739 | 1,525 | 3,222 | 3,571 | 4,144 | 4,417 | 3,064 | 1,118 | 182 |  |
| Endometrial cancer | | |  |  |  |  |  |  |  |  |  |  |  |  |  |  |  |  |  |
| Total | 0 | 1 | 13 | 39 | 88 | 208 | 366 | 520 | 622 | 587 | 702 | 881 | 726 | 781 | 688 | 420 | 158 | 18 |  |
| Men | 0 | 0 | 0 | 0 | 0 | 0 | 0 | 0 | 0 | 0 | 0 | 0 | 0 | 0 | 0 | 0 | 0 | 0 |  |
| Women | 0 | 1 | 13 | 39 | 88 | 208 | 366 | 520 | 622 | 587 | 702 | 881 | 726 | 781 | 688 | 420 | 158 | 18 |  |
| Dementia |  |  |  |  |  |  |  |  |  |  |  |  |  |  |  |  |  |  |  |
| Total | 0 | 0 | 0 | 0 | 0 | 0 | 3 | 11 | 37 | 148 | 404 | 1,353 | 2,908 | 6,436 | 11,849 | 13,156 | 7,267 | 1,730 |  |
| Men | 0 | 0 | 0 | 0 | 0 | 0 | 2 | 8 | 25 | 97 | 281 | 833 | 1,683 | 3,262 | 4,841 | 3,692 | 1,281 | 143 |  |
| Women | 0 | 0 | 0 | 0 | 0 | 0 | 1 | 3 | 12 | 51 | 123 | 520 | 1,225 | 3,174 | 7,008 | 9,464 | 5,986 | 1,587 |  |
| Depression |  |  |  |  |  |  |  |  |  |  |  |  |  |  |  |  |  |  |  |
| Total | 0 | 0 | 2 | 1 | 3 | 8 | 12 | 11 | 22 | 28 | 29 | 48 | 46 | 73 | 89 | 54 | 21 | 5 |  |
| Men | 0 | 0 | 2 | 0 | 2 | 5 | 7 | 6 | 11 | 18 | 19 | 27 | 18 | 21 | 16 | 13 | 4 | 0 |  |
| Women | 0 | 0 | 0 | 1 | 1 | 3 | 5 | 5 | 11 | 10 | 10 | 21 | 28 | 52 | 73 | 41 | 17 | 5 |  |
| Abbreviations: CVD, cardiovascular diseases  Data source: Ministry of Health, Labour and Welfare. Japanese Vital Statistics (January 1, 2021–December 31, 2021), Number of deaths of cause of death: by age group and sex ,https://www.e-stat.go.jp/stat-search/files?page=1&layout=datalist&toukei=00450011&tstat=000001028897&cycle=7&year=20210&month=0&tclass1=000001053058&tclass2=000001053061&tclass3=000001053065&result_back=1&tclass4val=0 [Accessed February 18 2025] | | | | | | | | | | | | | | | | | | | |
